# Thermal Characterization and Heat Capacities of Seven Polyphenols

**DOI:** 10.3390/molecules30010199

**Published:** 2025-01-06

**Authors:** Iván Montenegro, Carmen Pérez, Begoña González, Ángeles Domínguez, Elena Gómez

**Affiliations:** 1 FEQx Lab, Department of Chemical Engineering, University of Vigo, 36310 Vigo, Spain; ivan.montenegro@uvigo.gal (I.M.); bgp@uvigo.es (B.G.); admguez@uvigo.es (Á.D.); 2 CINTECX, ENCOMAT Group, University of Vigo, 36310 Vigo, Spain; cperez@uvigo.gal

**Keywords:** polyphenols, DSC, TGA, heat capacity, enthalpy of fusion, decomposition

## Abstract

Polyphenolic compounds are key elements in sectors such as pharmaceutics, cosmetics and food; thus, their physicochemical characterization is a vital task. In this work, the thermal behavior of seven polyphenols (*trans*-resveratrol, *trans*-polydatin, kaempferol, quercetin, myricetin, hesperidin, and (−)-epicatechin) was investigated with DSC (differential scanning calorimetry) and TGA (thermogravimetric analysis). Melting temperatures, enthalpies of fusion and decomposition temperatures were determined, and heat capacities were measured in the temperature range from 283.15 K to 363.15 K. Results were compared to the scarce experimental data available in the literature, showing a satisfactory agreement. All compounds were found to be thermally stable until melting, upon which they rapidly decomposed. Myricetin was the only polyphenol that presented polymorphic behavior, exhibiting two phase transitions prior to melting. Heat capacities increased minimally with temperature in the studied range. In addition, the group contribution method developed by Marrero and Gani was used to estimate the thermal properties of the polyphenols, achieving high accuracy for melting temperatures.

## 1. Introduction

Polyphenols are bioactive compounds present in numerous plant species. The strong antioxidant character of these multifunctional molecules grants them anti-tumor, anti-inflammatory, cardio-protective, anti-aging, anti-cancer, and anti-microbial properties, among others [1,2,3,4,5,6,7,8]. Due to all these features, they have garnered considerable attention in relevant sectors such as food, cosmetics, medicine, bio- and nanotechnology and pharmaceutics [8,9,10,11,12,13,14,15], and thus numerous lines of research have focused on the study of polyphenols in the past decade. Specifically, the extraction of antioxidants from organic residues of fruits with high polyphenolic content—such as grapes and apples—is proving to be very promising due to its potential contribution to circular economy and waste revaluation [16,17,18,19,20,21,22].

Structurally, polyphenols can be divided into non-flavonoids (mainly stilbenes and phenolic acids) and flavonoids (flavanols, flavonols, flavanones, flavones, and anthocyanins). The latter can be further sub-classified depending on the saturation and oxidation degrees of the carbonic ring, and each of them has a glycoside form with an attached sugar molecule; in addition, different combinations of functional groups (kaempferol, quercetin, myricetin) and isomers (catechin and epicatechin) exist for a given polyphenolic sub-class [23]. As a result, the number of known polyphenols exceeds 8000 [24], thus representing a broad and complex field of research to cover.

Against this background, thermal characterization is essential for the comprehensive study of any compound. It provides crucial information about phase transitions, decomposition temperatures and thermal properties, e.g., melting points and enthalpies of fusion, which are indispensable for the optimal design of extraction and purification processes and fortified food treatment. In pharmaceutics, the thermal characterization of drugs allows the stability analysis and identification of compounds, as well as activity and purity determination and selection of packaging materials. In addition, heat capacities are required for physicochemical processing and calculation of thermodynamic potentials, which is necessary for thermodynamic modeling and analysis of multicomponent systems. Nevertheless, for most polyphenols, the study of these properties has been merely superficial so far.

Therefore, in this work, the thermal behavior of seven polyphenols was analyzed and their heat capacities in the solid state were determined from 283.15 K to 363.15 K. Melting points and enthalpies of fusion were obtained via differential scanning calorimetry (DSC), whereas decomposition temperatures were determined by thermogravimetric analysis (TG). The selection of compounds was carried out considering their concentration in plant species, the polyphenolic division and industrial application. This way, seven representative polyphenols of grape waste were studied [25] (chemical structures are displayed in Figure S1): two stilbenes (*trans*-resveratrol and *trans*-polydatin), three flavonols (kaempferol, quercetin, and myricetin), one flavanone (hesperidin), and one flavanol ((−)-epicatechin). Experimental melting temperatures and enthalpies of fusion were compared to the scarce literature data available and also to predicted values calculated by the group contribution method developed by Marrero and Gani [26] for thermal properties. As for heat capacities, no data were found except for quercetin.

## 2. Results and Discussion

### 2.1. Thermal Analysis

#### 2.1.1. Experimental Results

**Figure 1:**
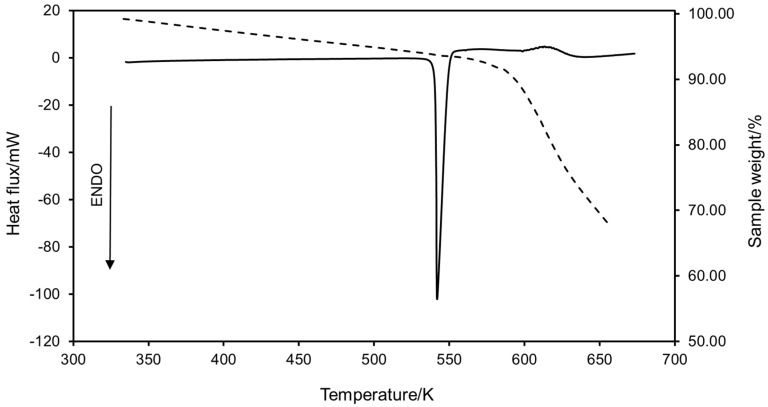
DSC (**—**) and TG (---) curves of *trans*-resveratrol.

The thermal behavior of seven polyphenols was evaluated using DSC and TG techniques. Figure 1 shows the DSC and TG curves of *trans*-resveratrol as an example (see Supplementary Information for the rest of curves), and experimental melting temperatures and enthalpies of fusion are included in Table 1, along with literature data for comparison. To perform the analysis, deviations from reference data are defined as follows:

(1) $$\mathrm{ARD}=\left|\frac{X_{\mathrm{EXP},i}\;-\;X_{\mathrm{REF},j}}{X_{\mathrm{REF},j}}\right|\times 100$$

(2) $$\mathrm{AARD}=\left(\sum_{j=1}^{n}\left(\frac{X_{\mathrm{EXP},i}\;-\;X_{\mathrm{REF},j}}{X_{\mathrm{REF},j}}\right)\times 100\right)/n$$

where ARD is the absolute relative deviation of any experimental magnitude X_EXP,i_ determined in this work from any literature reference X_REF,j_ for a given compound i, AARD is the average absolute relative deviation among j referenced values and n is the number of the selected literature references for each polyphenol.

All polyphenols proved to be thermally stable until melting, which is represented by a sharp endothermic peak for all compounds, indicating their crystalline nature. A clear exothermic peak is exhibited after melting in the DSC curves, which was accompanied by strong weight loss in the TG curves. This behavior could be associated with the degradation of the compounds. In the case of quercetin and hesperidin, this process took place immediately after the melting process, whereas *trans*-resveratrol and kaempferol (see Figure S3) remained stable around 60 K above their melting points, and so did *trans*-polydatin for almost 40 K after fusion. As for myricetin, melting and decomposition occurred almost simultaneously. The case of (−)-epicatechin stood out, as an exothermic peak did not appear, and vigorous degradation was observed after the fusion, representing the carbonization of the compound.

A clear trend in the melting temperatures of flavonols was noticed, which increased with the number of hydroxyl groups present in the aromatic ring in the following order: kaempferol, quercetin, myricetin. In all cases, small weight loss, between 2 and 3% depending on the flavonol, was observed around 373 K, related to water evaporation. In addition, two solid–solid state transitions (SST) were identified for myricetin at T_SST1_ = 565.80 K (ΔH_SST1_ = 24.99 J·g^−1^) and T_SST2_ = 612.15 K (ΔH_SST2_ = 4.19 J·g^−1^), which had no associated mass loss. This polymorphic behavior was also reported in previous studies [27,28] and might be the cause of the shortened melting peak (see Figure S5) and abnormally low enthalpy of fusion of myricetin (ΔH_m_ = 81.26 J·g^−1^) compared to those of the other two flavonols studied.

As for the melting temperatures, an excellent agreement with the scarce literature data was achieved in all cases. Maximum ARDs of 3.08%, 1.77%, and 1.52% were obtained for (−)-epicatechin, *trans*-polydatin, and myricetin, respectively, while for the rest of polyphenols, they only differ by up to 0.66%. Enthalpies of fusion are also very consistent with those previously reported, deviating 10.29%, 8.01%, and 1.47% on average (AARD) for hesperidin, (−)-epicatechin, and *trans*-resveratrol, respectively. Regarding kaempferol, a high discrepancy with the values obtained by Danciu et al. [29] was encountered, which was probably due to the small mass of the sample used for DSC in their study (mass = 2.53 mg). The melting enthalpy of quercetin is within the range of results obtained in the literature, and, to the best of our knowledge, no data were found for myricetin and *trans*-polydatin.

**Table 1:** Experimental melting temperatures, T_m_, and enthalpies of fusion, ΔH_m_, of the studied polyphenols, along with literature values.

Compound	T_m_/K		$\Delta H_{m}/J\cdot g^{-1}$	
	Exp.	Ref.	Exp.	Ref.
*Trans*-resveratrol	541.95	541.25 [30], 542.25 [31], 541.66 [32]	294.53	303.20 [33], 293.6 [34], 298.2 [35]
*Trans*-polydatin	504.65	503.15 [36], 501.15 [37], 495.85 [38]	187.93	No data found
Kaempferol	557.51	558.15 [39], 561.23 [40], 560.15 [41]	163.68	34.45 [29]
Quercetin	596.49	596.15 [42], 596.15 [43], 596.25 [44]	176.96	155 [43], 206.6 [45], 115.37 [46]
Myricetin	638.75	646.15 [28], 641.15 [27], 629.21 [47]	81.26	No data found
Hesperidin	532.77	531.15 [42], 532.15 [48], 533.15 [49]	120.76	122 [42], 101.24 [50]
(−)-Epicatechin	524.84	512.15 [51], 520.32 [52], 509.15 [53]	150.78	139.6 [52]

Regarding the decomposition temperatures extracted from the TG curves, Table 2 shows the experimental values together with data from the literature. As with the melting temperatures, the values obtained are in agreement with the experimental references, with AARDs between 0.024% and 0.98% for *trans*-polydatin, myricetin, hesperidin, and (−)-epicatechin. Concerning *trans*-resveratrol and quercetin, higher discrepancies were noticed due to a remarkable divergence among the literature values, achieving AARDs of 2.70% and 7.28%, respectively. Kaempferol presents the highest deviation, with an 11.19% ARD with respect to the only one found in the literature, obtained by Cao et al. [54].

#### 2.1.2. Property Prediction by Group Contribution Method

The melting temperatures and enthalpies of fusion of the seven polyphenols were calculated by the group contribution method developed by Marreno and Gani [26], which has been widely employed for the estimation of thermal properties. The results are displayed in Table 3, as are the relative deviations from our experimental values regarding melting points (ARD_Tm_) and enthalpies of fusion (ARD_ΔHm_), which are redefined for this section, as shown in Equations (3) and (4):

(3) $$\mathrm{ARD}=\left|\frac{X_{\mathrm{GC},i}-X_{\mathrm{EXP},i}}{X_{\mathrm{EXP},i}}\right|\times 100$$

(4) $$\mathrm{AARD}=\left(\sum_{i=1}^{n}\left(\frac{X_{\mathrm{GC},i}-X_{\mathrm{EXP},i}}{X_{\mathrm{EXP},i}}\right)\times 100\right)/n$$

where X_GC,i_ refers to a thermal property of a certain polyphenol i, estimated with the group contribution method provided by Marreno and Gani [26], while n indicates the number of polyphenols.

The resulting AARD for melting temperatures is 9.05%, denoting an adequate estimation. Predicted values diverge by less than 5% from experimental results for *trans*-polydatin and (−)-epicatechin and up to 10% for kaempferol, hesperidin, and quercetin. On the contrary, for *trans*-resveratrol and myricetin, this threshold is slightly exceeded. Regarding flavonols, it is also observed that the ARDs in the experimental values increase with the molecular weight, i.e., with the number of hydroxyl groups present in the aromatic rings. This fact highlights the influence of the position of the hydroxyl group in the benzene ring, which is not considered in the group contribution method applied. As for the enthalpy of fusion, the best estimation was again achieved for *trans*-polydatin, this being below 5% of ARD, whilst moderate discrepancies (<14%) were obtained for kaempferol, quercetin, and (−)-epicatechin. The accuracy of the method was insufficient for *trans*-resveratrol, hesperidin, and myricetin, with the latter achieving the greatest ARD compared to our experimental results. This is probably due to the near-fusion polymorphism of the compound, a phenomenon not accounted for by the group contribution method, and is probably the reason why deviations are remarkably lower for the other two flavonols.

### 2.2. Heat Capacities

**Figure 2:**
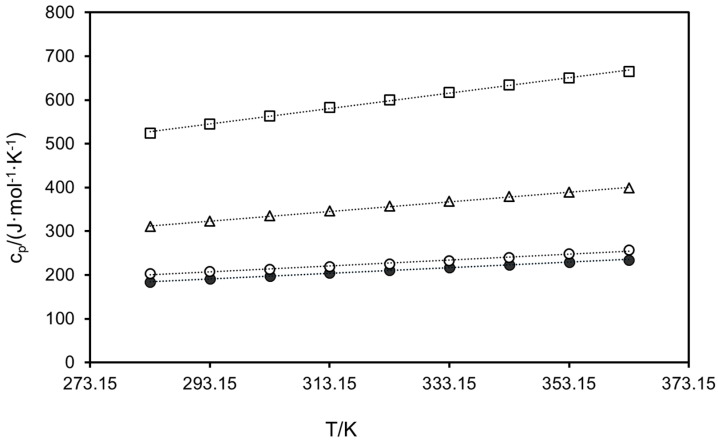
Experimental molar heat capacities of (●) *trans*-resveratrol, (**△**) *trans*-polydatin, (□) hesperidin, and (○) (−)-epicatechin. Dotted lines are depicted for better trend visualization.

**Figure 3:**
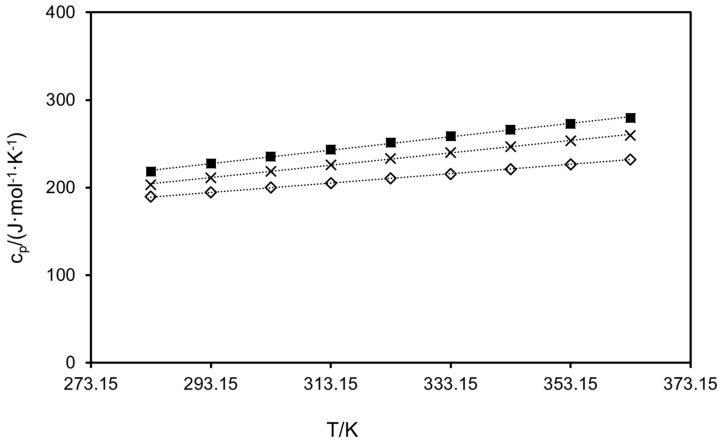
Experimental molar heat capacities of (⨉) kaempferol, (◇) quercetin, and (■) myricetin. Dotted lines are depicted for better trend visualization.

The heat capacities of the seven compounds were measured via DSC following the sapphire method in a three-stage program from 273.15 K to 373.15 K. Values below 283.15 K and above 363.15 K were discarded for accuracy reasons. Results in 10 K steps are plotted in Figure 2 and Figure 3, and were linearly fitted by Equation (5):

(5) $$c_{p}=y_{0}+a\times \;T(K)$$

where c_p_ is the heat capacity in J·g^−1^·K^−1^, and y_0_ and a are fitting coefficients. The latter, along with the R^2^ values obtained from the regression, are collected in Table 4 for each polyphenol. Although the calculation of heat capacities was performed on a mass basis (J·g^−1^·K^−1^) as dictated by the procedure, molar heat capacities (J·mol^−1^·K^−1^) are displayed in Figure 2 and Figure 3 for purely comparative purposes, since this property is roughly additive and molar values are recommended for the analysis regarding structural features of the polyphenols.

Slight increases with temperature are observed for all compounds in the range studied, the most notorious being for trans-polydatin (28.31%) and the least being for quercetin (22.39%), although slopes from the fitting are quite similar in all cases. Hesperidin achieved higher heat capacities in the whole temperature range than any other polyphenol, with a maximum of 665.66 J·mol^−1^·K^−1^ at 363.15 K, while the minimum corresponds to *trans*-resveratrol, with 183.66 J·mol^−1^ K^−1^ at the lowest temperature. This highlights the trend of the molar heat capacities with the molecular weight of the compounds, as the heavier the compound, the higher the molar heat capacity, following the order: hesperidin > *trans*-polydatin > myricetin > (−)-epicatechin ≅ kaempferol > *trans*-resveratrol. Quercetin is the only compound that does not follow the trend, differing with *trans*-resveratrol by 1.09% on average in the studied range. As for (−)-epicatechin and kaempferol, they differ by only 2.37% on average in the whole temperature range, which is in agreement with their almost equal molecular weights (290.27 g·mol^−1^ and 286.24 g·mol^−1^, respectively).

As far as stilbenes are concerned, the molar heat capacities of *trans*-polydatin are 1.70 times higher than those of *trans*-resveratrol from 283.15 K to 363.15 K, revealing the influence of the glucose molecule in the chemical species. Contrary to the melting temperatures, no trend was observed in Figure 3 regarding the number of hydroxyl groups of the flavonols; thus, kaempferol heat capacities were from 6.95% to 11.70% higher than those of quercetin in the temperature range studied, whereas values of myricetin exceeded those of kaempferol by 7.72% on average. These discrepancies emphasize that not only does the number of the hydroxyl group in the benzene ring influence the heat capacity, but so does its position.

No experimental data were found in the literature for any polyphenol except for quercetin. Li et al. [62] measured the heat capacity of pure quercetin from 298.2 K to 470.2 K. They also applied the three-step method with DSC, employing sapphire as the reference substance. However, they used a remarkably lower sample mass than we did (of about 5 mg, whereas the recommended amount is ten times higher), and a heating rate of 5 K min^−1^ for the dynamic segment. Average deviations of 44.45% were obtained with respect to their values in the temperature range from 298.2 K to 363.2 K.

## 3. Materials and Methods

### 3.1. Materials

Detailed information about the polyphenols used in this study is reported in Table 5. All of them were dried in situ before each experiment.

### 3.2. Methods

#### 3.2.1. Thermal Analysis

##### Melting Point and Enthalpy of Fusion

Thermal profiles of all polyphenols were determined by differential scanning calorimetry using a Mettler Toledo 822e/400 DSC apparatus. Samples of 4 to 12 mg were weighted on an AX-205 DeltaRange balance from Mettler Toledo (Columbus, OH, USA) and placed in 40 µL aluminum crucibles with a pinhole. Measurements were carried out covering the melting of all compounds as follows: performing dynamic heating from 298.15 K to 673.15 K at a heating rate of 10 K·min^−1^, with previous drying at 393.15 K for 15 min. Inert atmosphere was maintain throughout the whole study with a 50 mL·min^−1^ constant flow of nitrogen. Melting temperatures were considered the endothermic peaks of the DSC curves, whereas enthalpies of fusion were directly integrated with the apparatus software STAR^e^ SW 16.10. Experiments were performed in duplicate for each polyphenol.

Calibration for temperature and heat flow was performed using pure zinc, indium, water, and heptane, with a repeatability in the enthalpy of fusion of ±2% and in the temperature of ±0.2%. To estimate the uncertainty in the measurement of temperature, a pure-indium sample (provided by Mettler Toledo) was used. The following variables were taken into account:

(i) Uncertainty of apparatus: Three replicate scans of the same sample were completed in the furnace. This uncertainty was found to be ±0.03 K for melting temperature and ±0.5 J·g^−1^ for enthalpy of fusion.

(ii) Uncertainty of the operator and positioning in the furnace: Two different operators removed and replaced the sample three times each. The uncertainty was ±0.35 K for melting temperature and ±1.5 J·g^−1^ for enthalpy of fusion.

(iii) Uncertainty of sample and pan: A new pan with pure indium was used. This uncertainty was found to be ±0.05 K for melting temperature and ±0.6 J·g^−1^ for enthalpy of fusion.

Finally, in this work, the standard uncertainty in the measurement of the temperature and in the enthalpy of fusion were assumed to be ±1 K for melting temperature and ±2 J·g^−1^ for enthalpy of fusion, since the phase transition temperature can depend on other variables, such as the heating and cooling rate.

#### 3.2.2. Heat Capacity

The classical three-step procedure [63] was chosen for heat capacity determination using DSC, employing sapphire (ref. ME-51140818) as the reference substance. It consisted of three segments: the first isothermal period at 273.15 K for 15 min, followed by dynamic heating to 373.15 K at 10 K·min^−1^, and ending with a final isothermal segment for other 15 min at 373.15 K. Pre-drying was performed at 393.15 K for 15 min. A constant nitrogen flow of 50 mL·min^−1^ was supplied to the furnace. Since this is a comparative procedure, a higher amount of sample (≅50 mg) was necessary for each experiment, as the measurement signal provided by both the sapphire and the sample must be clear and sufficiently close to attain high accuracy [64]. An AX-205 DeltaRange balance from Mettler Toledo was again used for the weighting process for the samples. Measurements were performed in duplicate for each polyphenol.

To calculate the uncertainty in the measurement of heat capacity, a pure sapphire sample (supplied by Mettler Toledo) was used. The same variables for the estimation of the uncertainty in the measurement of temperature and enthalpy of fusion were taken into account:

(i) Uncertainty of apparatus: This uncertainty was found to be ±0.00013 J·K^−1^·g^−1^.

(ii) Uncertainty of the operator and positioning in the furnace: This uncertainty was ±0.0035 J·K^−1^·g^−1^.

(iii) Uncertainty of sample and pan: A new pan with pure sapphire was used; this uncertainty was found to be ±0.0015 J·K^−1^·g^−1^.

In this case, taking into account the influence of the uncertainty of the mass of the sample and of the sapphire reference on the overall uncertainty of the measurement of heat capacity, the standard uncertainty was estimated to be ±0.006 J·K^−1^·g^−1^.

#### 3.2.3. Thermogravimetric Analysis

TG measurements were carried out using a Mettler Toledo TGA/DSC1 instrument. Compounds were used as supplied, with no purification method applied. Powder samples, between 9 and 20 mg in weight, were placed in 40 μL pinhole aluminum crucibles. They were heated from 298 K to 723 K at a heating rate of 10 K·min^−1^, and a nitrogen stream with a flow of 50 mL·min^−1^ was used. Decomposition temperatures were considered the onset of the TG curves, from which the mass lost was considerable. This procedure was carried out in duplicate for each polyphenol.

## 4. Conclusions

The thermal behavior of seven polyphenols present in plant waste was investigated through DSC and TGA. Melting points, enthalpies of fusion, and decomposition temperatures were determined, and heat capacities were calculated from 283.15 K to 363.15 K; none had been reported so far in the literature for most compounds studied.

All compounds proved to be thermally stable until fusion, upon which decomposition started. Myricetin stands out for being the only polyphenol that shows polymorphic behavior, exhibiting two solid–solid transitions close to melting. Good agreement with the literature values was observed, despite the scarce experimental data available. In addition, the group contribution method developed by Marrero and Gani [26] was used to predict the thermal properties of the polyphenols, having been proven to be able to estimate the melting temperatures of all compounds with high accuracy. In addition, heat capacities showed a slight increase with temperature in the range studied for all cases, and a clear upward trend with the molecular weight was observed for this thermophysical property in all polyphenols except for quercetin.

## Figures and Tables

**Table 2:** Comparison of experimental decomposition temperatures (T_d_) with referenced values.

Compound	T_d_/K	
	Exp.	Ref.
*Trans*-resveratrol	593.25	511.45 [55], 546.15 [56], 563.75 [34]
*Trans*-polydatin	542.58	543.15.15 [57], 553.15 [58], 546.15 [37]
Kaempferol	631.97	561.25 [54]
Quercetin	623.58	609.15 [42], 594.15 [59], 617 [60]
Myricetin	633.30	633.15 [61]
Hesperidin	553.6	555.15 [42], 539.15 [54]
(−)-Epicatechin	524.84 ^a^	521.15 K [52]

^a^ Melting and decomposition occurred simultaneously.

**Table 3:** Comparison of experimental melting points and enthalpies of fusion (T_m_, ΔH_m_) and those calculated by the group contribution method developed by Marrero and Gani [26] (T_m,GC_, ΔH_m,G.C_).

Compound	T_m_/K	T_m,GC_/K	ARD_Tm_/%	ΔH_m_/J·g^−1^	ΔH_m,GC_/J·g^−1^	ARD_ΔHm_/%
*Trans*-resveratrol	541.95	486.89	10.16	294.53	201.52	31.58
*Trans*-polydatin	504.65	525.15	4.06	187.93	194.03	3.24
Kaempferol	557.51	525.40	5.76	163.68	145.78	10.93
Quercetin	596.49	538.24	9.77	176.96	154.39	12.76
Myricetin	638.75	557.55	12.71	81.26	160.88	97.98
Hesperidin	532.77	582.71	9.37	120.76	159.35	31.96
(−)-Epicatechin	524.84	512.01	2.44	150.78	170.56	13.12

**Table 4:** Fitting parameters of the linear regression of experimental c_p_ (J·g^−1^·K^−1^).

Compound	y_0_	a·10^3^	R^2^
*Trans*-resveratrol	0.0181	2.80	0.998
*Trans*-polydatin	0.0038	2.81	0.998
Kaempferol	0.0158	2.46	0.998
Quercetin	0.1305	1.75	0.999
Myricetin	0.0134	2.39	0.998
Hesperidin	0.0456	2.89	0.998
(−)-Epicatechin	0.0318	2.33	0.990

**Table 5:** Information of materials used for experimentation.

Compound	CAS Number	Source	Molecular Weight/g·mol^−1^	Mass Purity ^a^/%
*Trans*-resveratrol	501-36-0	TCI	228.24	>99.0
*Trans*-polydatin	65914-17-2	Sigma-Aldrich	390.38	≥95
Kaempferol	520-18-3	Apollo Scientific	286.24	98
Quercetin	117-39-5	Sigma-Aldrich	302.24	≥95
Myricetin	529-44-2	Sigma-Aldrich	318.24	>97.0
Hesperidin	520-26-3	Supelco	610.57	≥99.9
(−)-Epicatechin	490-46-0	TCI	290.27	≥97.0

^a^ Provided by the supplier.

## Data Availability

Data are contained within the article or Supplementary Material. The original contributions presented in this study are included in the article/Supplementary Material. Further inquiries can be directed to the corresponding authors.

## Supplementary Materials

The following supporting information can be downloaded at: https://www.mdpi.com/article/10.3390/molecules30010199/s1. Figure S1: Chemical structure of *trans*-resveratrol (a), *trans*-polydatin (b), kaempferol (c), quercetin (d), myricetin (e), (−)-epicatechin (f), and hesperidin (g). Figure S2: DSC (—) and TG (---) curves of *trans*-polydatin. Figure S3: DSC (—) and TG (---) curves of kaempferol. Figure S4: DSC (—) and TG (---) curves of quercetin. Figure S5: DSC (—) and TG (---) curves of myricetin. Figure S6: DSC (—) and TG (---) curves of hesperidin. Figure S7: DSC (—) and TG (---) curves of (-)-epicatechin.
