# Supplementary material for: Thermal Characterization and Heat Capacities of Seven Polyphenols
Source: Molecules. 2025 Jan 6;30(1):199. doi: 10.3390/molecules30010199 (PMC11722974; doi:10.3390/molecules30010199)
Supplement: Supplementary file 1 [file molecules-30-00199-s001.zip › molecules-3377527-supplementary.pdf]

SUPPLEMENTARY MATERIAL

Thermal Properties of Pharmaceutical Antioxidants: Heat Capacities and Melting Temperatures

Molecules

Iván Montenegro[a], Carmen Pérez[b], Begoña González[a], Ángeles Domínguez[a], Elena Gómez[a,*]

[a]FEQx lab, Department of Chemical Engineering, University of Vigo, Spain
[b]CINTECX, ENCOMAT Group, Material Science Department, CINTECX, University of Vigo, Spain

* Corresponding author
E-mail address: elenagc@uvigo.es

Figure Index

Figure S1 Chemical structure of *trans*-resveratrol, *trans*-polydatin, kaempferol, quercetin, myricetin, (-)-epicatechin, and hesperidin 2

Figure S2 DSC and TG curves of *trans*-polydatin 3

Figure S3 DSC and TG curves of kaempferol 3

Figure S4 DSC and TG curves of quercetin 4

Figure S5 DSC and TG curves of myricetin 4

Figure S6 DSC and TG curves of hesperidin 5

Figure S7 DSC and TG curves of (-)-epicatechin 5

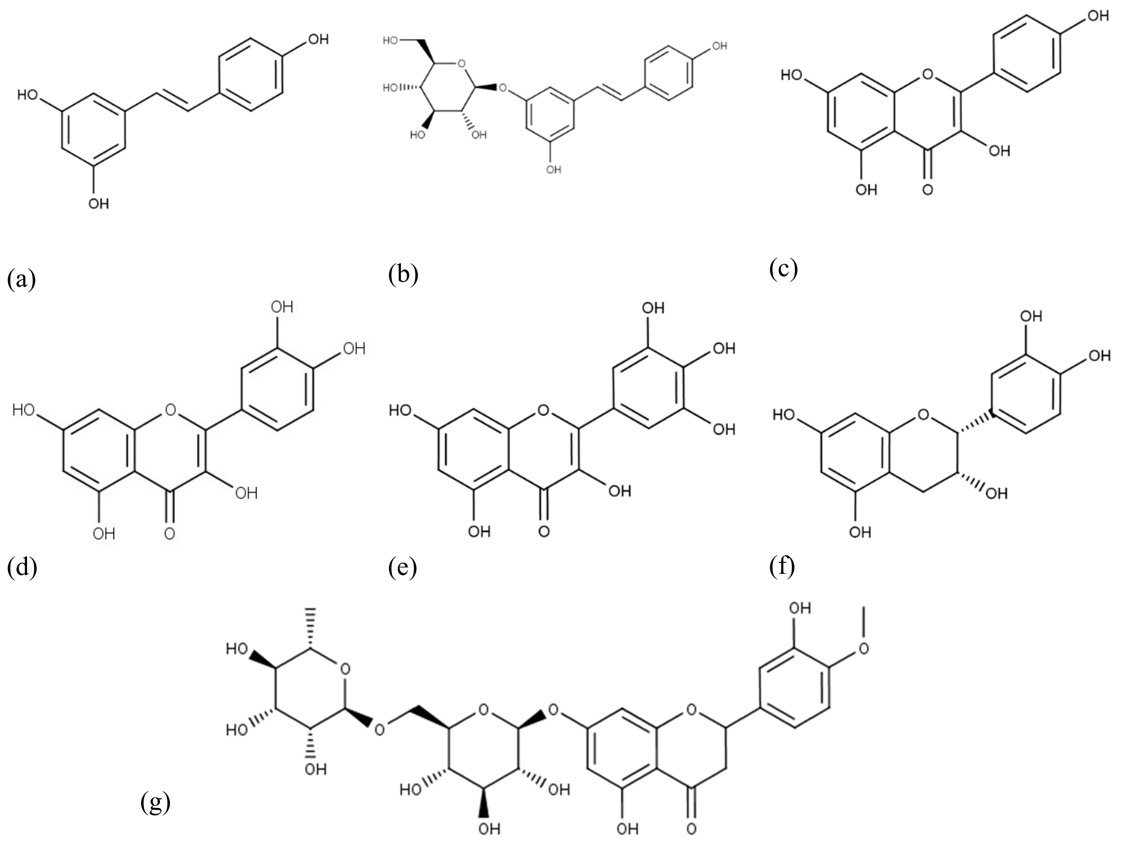

Figure S1 Chemical structure of *trans*-resveratrol (a), *trans*-polydatin (b), kaempferol (c), quercetin (d), myricetin (e), (-)-epicatechin (f), and hesperidin (g)

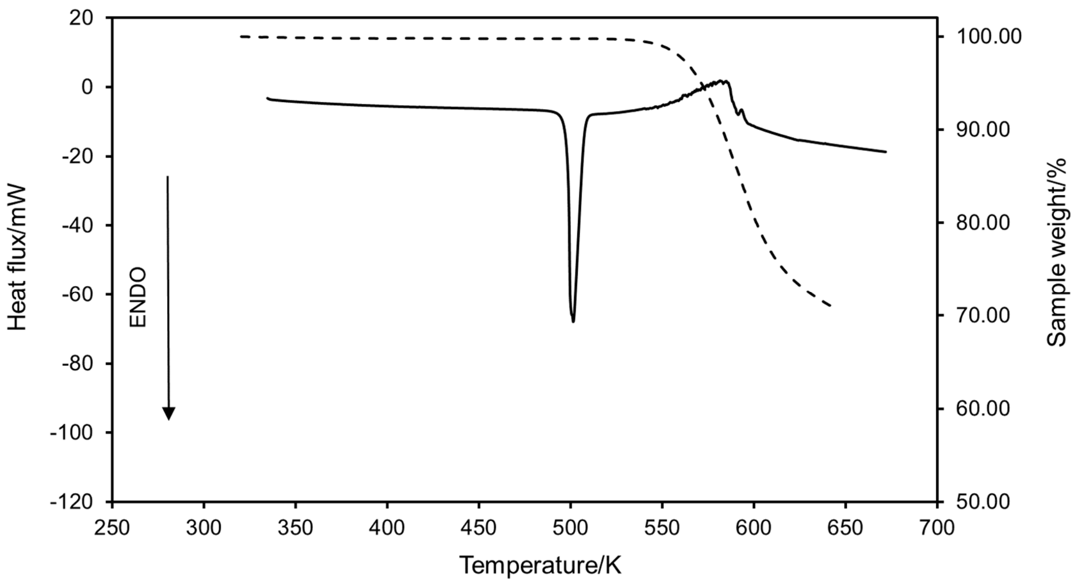

Figure S2 DSC (—) and TG (---) curves of *trans*-polydatin

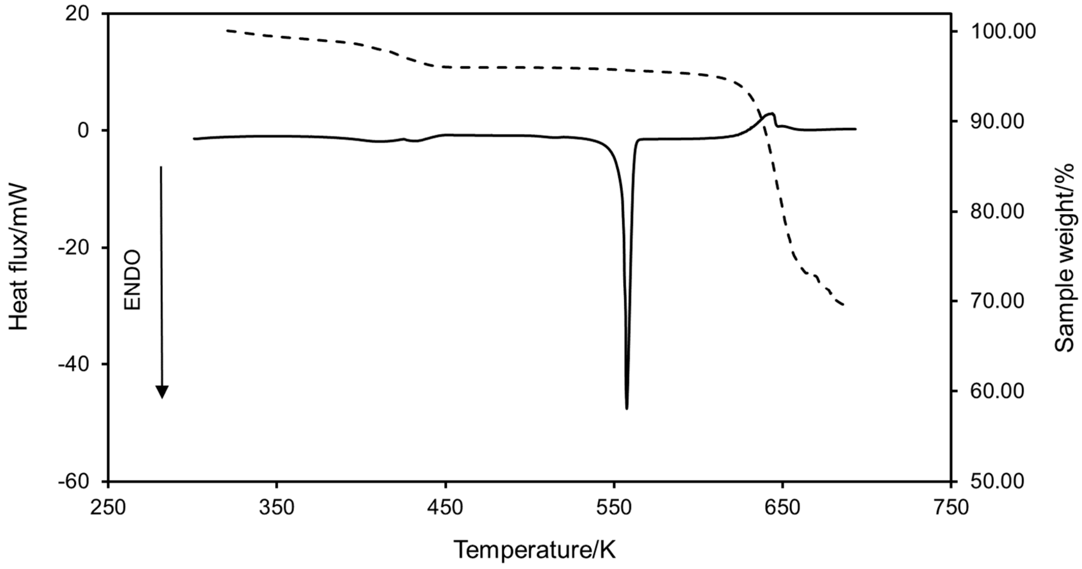

Figure S3 DSC (—) and TG (---) curves of kaempferol

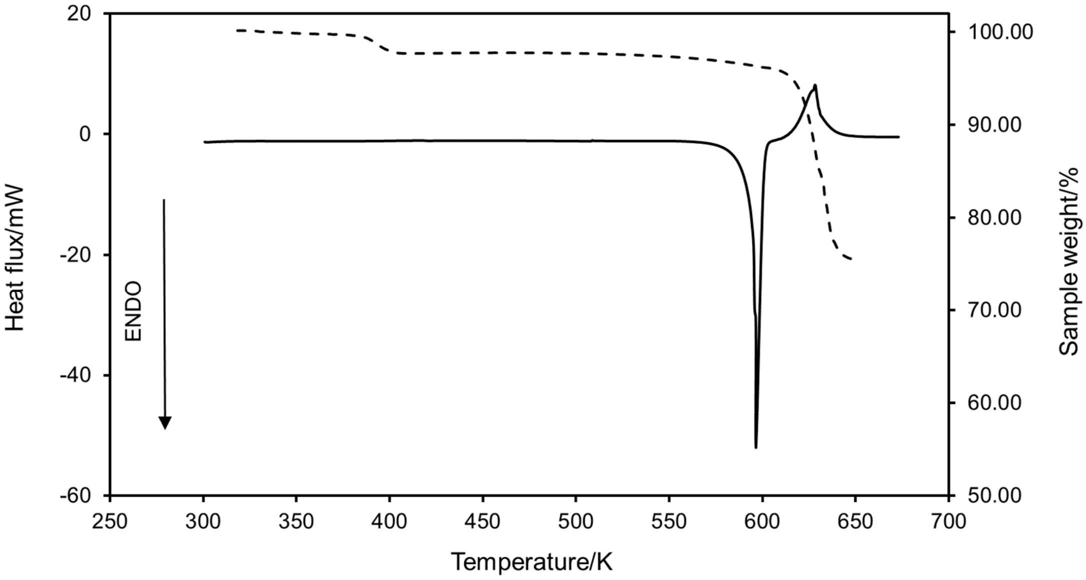

Figure S4 DSC (—) and TG (---) curves of quercetin

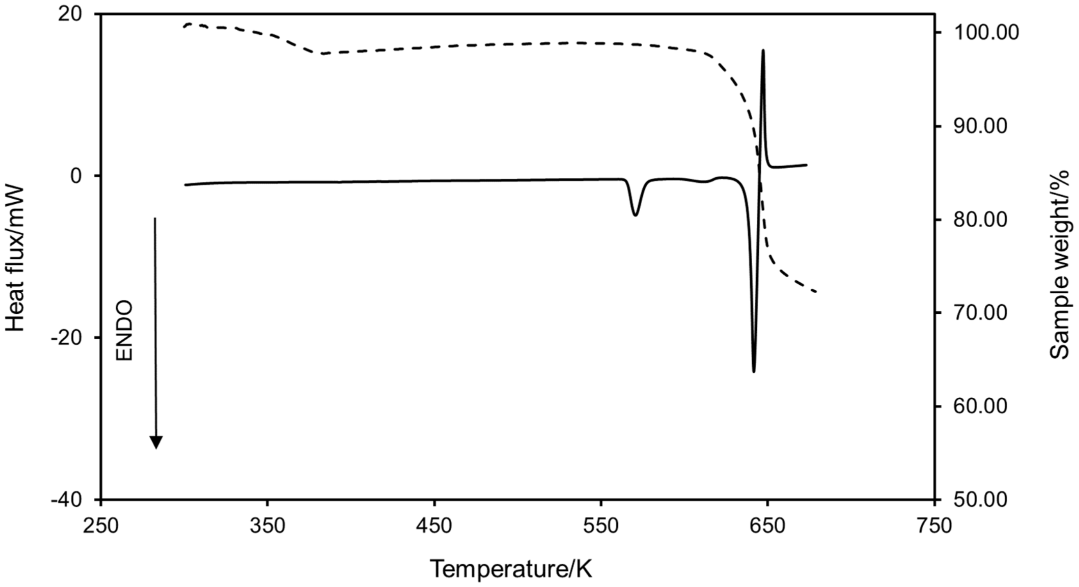

Figure S5 DSC (—) and TG (---) curves of myricetin

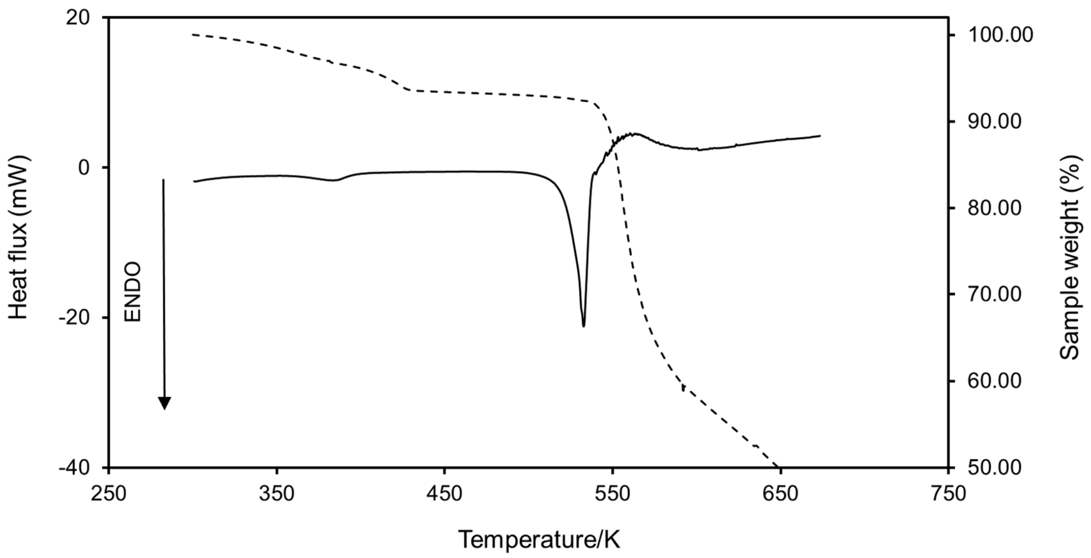

Figure S6 DSC (—) and TG (---) curves of hesperidin

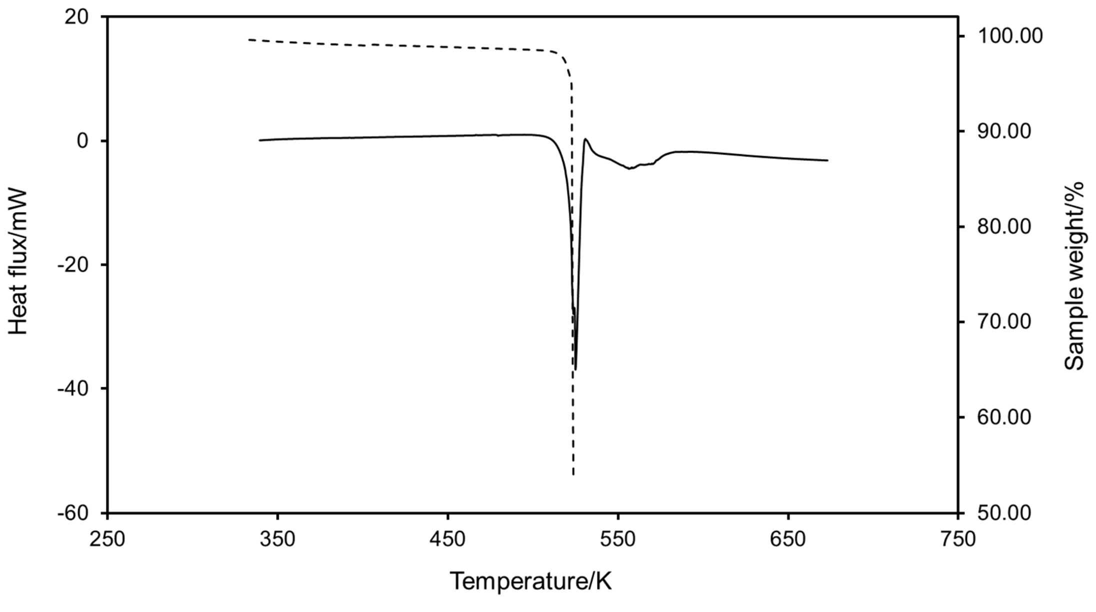

Figure S7 DSC (—) and TG (---) curves of (-)-epicatechin
